# Our Bureau of Information

**Published:** 1912-03-23

**Authors:** 


### OUR BUREAU OF INFORMATION.

### Milk and Babies.

THE price of milk has gone down by a penny the quart. The statement is one that concerns national health as well as national trade. Our civilisation seems to be lessening the supply of mothers' milk in every rank of life. It has been the fashion to talk as if the refusal of a mother to nurse her child was confined to those circles where "social duties" are supposed to rule. But industrial necessity is as potent a factor in artificial feeding, and unfortunately where this regulates the mother's life good artificial feeding is seldom to be found. Mothers in this class prefer to suckle their children; it saves the trouble (no slight matter to a weary woman) of getting up in the night to prepare bottles. But if the mother must go out to work, factory-work, laundry-work, and even charing, though in a less degree, tend to lessen the supply of milk and injure the quality. Hence much artificial feeding among the working-classes, and artificial feeding of the worst sort. The milk is bought from the nearest dairy, it is "modified" in the most casual manner to meet the supposed needs of the child, it may be administered either too hot or too cold, and it is almost invariably given through a long-tubed bottle of a doubtful cleanliness. And from this come results which are always serious and may be fatal. It may truthfully be said that among the poorer classes nine-tenths of the ailments from which babies suffer are due to malnutrition, and that in the majority of cases this is due to bad milk.

About a score of municipalities have milk depôts where poor women can obtain milk, guaranteed as pure, and modified to suit the various ages of young children. These have doubtless done much to save life and to educate mothers in the different results of using milk which has been tested and properly prepared, and milk which has no such guarantee. But of special organisations to meet the needs of babies what have we? There is an Infants' Hospital in Westminster with fifty beds, where children under eighteen months old are received and treated, and where those who interest themselves in the endless problems of infant vitality and mortality may study them in characteristic examples. And there is an Infants' Convalescent Home at Hunstanton, where children just saved from death may regain the vigour which they ought to have. We know of no special hospital for infants in the rest of England, though doubtless all children's hospitals get their share of these delicate blossoms of humanity. But are there other infant sanatoria? There is certainly room for them, but we can understand that they are not easy to establish. Apart from the initial cost, the working of them requires a body of women with the mother-heart—not always found in mothers—strongly developed, tireless devotion, and unfailing love. To expend these gifts on babies is not merely a kindness to the poor and helpless: it is a gift to the nation.

### Answers to Correspondents.

### VEGETARIAN DIET.

"VETERAN" writes: Do you believe in vegetarianism? I am fifty-five years of age and suffer a great deal with rheumatism. A friend who is a keen vegetarian tells me that if I stop eating meat I shall be all right.

[This is a matter on which you must consult your own medical man. He may think it wise to modify your diet, especially if you are a large meat-eater, but that is a point which can be settled only by someone who knows your case. But a sudden and entire change of food might cause considerable digestive disturbance, especially in a man of your age. It is a matter on which skilled advice is essential.]

### A HOSPITAL TREASURER'S SALARY.

"HALDON" writes: Some years ago I saw it stated that the treasurer of one of our London hospitals had a salary of £2,000 a year. People with moderate incomes are not going to subscribe to institutions whose officials draw such large salaries.

[The statement is absolutely false. Hospital treasurers are honorary officials, and receive no salary.]

### TRAINING SCHOOL IN THE SOUTH OF ENGLAND.

"DOROTHY R." writes: I am writing to ask if you could give me information as to nursing training to be obtained in some bracing provincial town in the south of England, preferably Brighton or Folkestone. I write on behalf of a friend, who is very anxious to take up nursing but is unable to live in London, and would find it difficult to afford a premium.

[Either the Sussex County Hospital, Brighton, or the Victoria Hospital, Folkestone, would meet your friend's requirements. The former is the larger institution. But she must remember that she must prove that her health is satisfactory if she wishes to enter any hospital. She will find all the information she wants in "How to Become a Nurse," edited by Sir Henry Burdett, obtainable from the Scientific Press, Southampton Street, W.C. It saves much trouble and disappointment when candidates know the requirements of the training school they wish to enter.]

### INDUSTRIAL TRAINING FOR DEFECTIVES.

"B. C. H." writes: Is there any industrial training given in schools for defective children?

[Only ordinary education is given to defective children up to the age of fourteen, but they can then be drafted for two years into senior schools, where they receive some industrial training.]

### SWISS HOSPITALS AND ENGLISH PROBATIONERS.

"E. E. C." writes: Could you give me addresses of hospitals in Switzerland where English probationers are taken?

["T. L." answers: Your correspondent should write to one of the following addresses: "Dr. Kraft, Directeur de l'Ecole de Garde-Malade, La Source, Lausanne"; "Direction de l'Hôpital de la Croix Rouge, Zurich"; or "Direction de l'Hôpital de la Croix Rouge, Berne."]

### Our List of Approved Homes.

We have pleasure in adding the following to our list of Approved Homes: Invalids' Home, 3 Milton Road, West Hartlepool. Principal, Sister Arnold. Medical, surgical, and chronic cases. Operating theatre. Bright convenient house, in quiet road near sea. Fees moderate. Sister Arnold also visits patients in their own homes.

### Rules for Correspondents.

Everyone who uses or wishes to help our Bureau of Information must be kind enough to observe the following rules:—

(1) Postal replies cannot be given. Exception will only be made in very special cases at the Editor's discretion.

(2) Queries or answers relating to different cases must be written, or preferably typed, on separate sheets of paper, and the writing must never be crossed.

(3) Questions and replies received not later than Wednesday will, as far as possible, be dealt with in our issue of the following Saturday week.

(4) Letters must have attached the name and address of the sender, with a pseudonym for publication if preferred.

(5) Applications referring to non-dependents where the need relates to business or employment must be accompanied by a postal order for 5s., when the requirement shall have publicity in these columns.

(6) All applicants for insertion in our list of Approved Homes must send a registration fee of five shillings. The omission of this leads to delay and gives avoidable trouble.

(7) *All communications* for this department *must be accompanied by the coupon* to be found at the bottom of the third page of the cover on the inside, and should be addressed to the Editor of the Bureau of Information, c/o THE HOSPITAL, 29 Southampton Street, Strand, London, W.C.

